# Knowledge Graph Completion for the Chinese Text of Cultural Relics Based on Bidirectional Encoder Representations from Transformers with Entity-Type Information

**DOI:** 10.3390/e22101168

**Published:** 2020-10-16

**Authors:** Min Zhang, Guohua Geng, Sheng Zeng, Huaping Jia

**Affiliations:** 1 School of Information Science and Technology, Northwest University, Xi’an 710127, China; zhangmin@stumail.nwu.edu.cn (M.Z.); shengzeng@stumail.nwu.edu.cn (S.Z.); 2 College of Computer, Weinan Normal University, Weinan 714099, China; jiahuaping_1979@126.com

**Keywords:** knowledge graph completion, cultural relics, link prediction, Bidirectional Encoder Representations from Transformers (BERT), entity type

## Abstract

Knowledge graph completion can make knowledge graphs more complete, which is a meaningful research topic. However, the existing methods do not make full use of entity semantic information. Another challenge is that a deep model requires large-scale manually labelled data, which greatly increases manual labour. In order to alleviate the scarcity of labelled data in the field of cultural relics and capture the rich semantic information of entities, this paper proposes a model based on the Bidirectional Encoder Representations from Transformers (BERT) with entity-type information for the knowledge graph completion of the Chinese texts of cultural relics. In this work, the knowledge graph completion task is treated as a classification task, while the entities, relations and entity-type information are integrated as a textual sequence, and the Chinese characters are used as a token unit in which input representation is constructed by summing token, segment and position embeddings. A small number of labelled data are used to pre-train the model, and then, a large number of unlabelled data are used to fine-tune the pre-training model. The experiment results show that the BERT-KGC model with entity-type information can enrich the semantics information of the entities to reduce the degree of ambiguity of the entities and relations to some degree and achieve more effective performance than the baselines in triple classification, link prediction and relation prediction tasks using 35% of the labelled data of cultural relics.

## 1. Introduction

Knowledge graphs (KGs) are typically multi-relational graphs representing entities and relationships, in which the nodes represent entities and the edges between the nodes represent the relations between the entities. One head entity h, one tail entity t, and a relation between the head and tail entities are represented as a triple (h,r,t). KGs have high-quality structured data and are the cornerstone of many artificial intelligence applications. In vertical fields such as finance and medical care, knowledge graphs bring better domain knowledge and perfect user experience [1]. In the field of cultural relics, KG technology can mine the relations among cultural relics and construct a knowledge database to effectively solve the problems related to the storage, display and management of cultural relics [2].

However, large-scale KGs, such as DBpedia and Freebase, which comprise millions of entities and hundreds of millions of relationships, are still far from complete. According to statistics, 60% of person entities lack a place of birth in DBpedia 2014, 75% of person entities are missing their nationalities, and 94% do not have facts about their parents in Freebase [3]. In the field of cultural relics, because of the particularity of word-formation methods for cultural relic entities, the implicit relations between them are difficult to explore. Regarding the cultural relic entity “Afterglow-style ‘Caifeng Mingqi’ seven-stringed guqin”, it was made in the Tang Dynasty and collected in the Zhejiang provincial museum. These explicit relations can be extracted using the relation extraction methods. However, it is hard to find implicit relations between “Afterglow-style ‘Caifeng Mingqi’ seven-stringed guqin” and “qishan”. Therefore, the cultural relic knowledge graph is also incomplete [4].

The objective of knowledge graph completion is to predict the missing part in a triplet so as to make the knowledge graph more complete. Considerable research has been devoted to knowledge graph completion or link prediction, which predicts the missing head/tail entities in an incomplete triple [5] and predicts whether a triple is valid [6]. Consequently, many studies have been conducted recently on how to use effective methods to better represent and complete KGs on this basis, so as to expand the scale of KGs.

Many embedding models using the vector or matrix representations of entities and relations to obtain the best link prediction results have been presented [7]. In these embedding models, the implausibility scores are used to predict the validity of triples. In many KGs, there are many low-connected entities. In this kind of sparse graph, only considering the structural information of triples increases the sparseness of the knowledge graphs, and the embedded information cannot be fully updated. In order to enrich the knowledge expression, some studies add textual information, but these methods ignore the contextual information and semantic information [8,9].

To improve this problem, many studies use deep models, e.g., the Convolutional Neural Network (CNN), Recurrent Neural Network (RNN) [10,11] and Capsule networks [12], to better capture contextual information and semantic features. These popular language representation models perform well in the knowledge graph completion task [13]. However, a deep learning-based model usually has a large number of parameters, which increases the model’s capacity but also introduces the risk of overfitting. In order to enhance the generalization ability of models, models based on deep learning usually need to be trained on large-scale labelled datasets. Unfortunately, it is time-consuming and laborious to obtain high-quality large-scale data on the text of cultural relics.

In recent years, pre-trained language models have obtained effective results in many natural language processing (NLP) tasks [14,15]. Examples include natural language inference and paraphrasing using Embeddings from Language Models (ELMo) [16] and Generative Pretraining (GPT) [17], which address the sentence-level tasks of predicting relations through sentences; meanwhile, named entity recognition and question answering using the Bidirectional Encoder Representation from Transformers (BERT) [18] address the token-level task of producing a fine-grained output. Particularly, BERT uses a transformer trained on larger-scale unlabelled data, which is more efficient than the RNN and can capture dependencies over longer distances. Compared with the previous pre-trained model, BERT can capture the richer bidirectional contextual information.

Meanwhile, BERT uses a large number of unlabelled data to pre-train the model and only uses a small number of labelled data to fine-tune the pre-trained model, which effectively captures the contextual information and performs well in multiple NLP tasks. Due to the lack of text-labelled data in the field of cultural relics, this paper utilizes BERT to complete the knowledge graph of cultural relics to address the problem of the lack of high-quality labelled data in the field of cultural relics. In addition, the Chinese text of cultural relics has strong contextual relevance, and the word formation of cultural relic entities has its particularity. The BERT language model is adopted to construct the cultural relic knowledge graph completion model for capturing the rich context information of the Chinese text of cultural relics.

Furthermore, there is a wealth of multisource information about entities and relations, e.g., the description information of entities and relations, the type information contained in the knowledge graph, and the massive online text information outside the knowledge graph. This multisource information provides additional information beyond the triple in the knowledge graph and helps to learn knowledge representations more accurately [2]. Particularly, as hidden variables, entity type information is crucial for reducing the degree of ambiguity of entities and relations and can be used as information supplementary to text to enrich the semantic information of an entity [19]. Therefore, this paper integrates entity type information as a part of the input of the BERT model to achieve more effective representations of entities.

In this study, a method based on the BERT language model with entity-type information is proposed for completing the knowledge graph of cultural relics. Especially, the entity-type information, entities, and relations are incorporated as textual sequences, and then, the knowledge graph completion task is turned into a classification problem; then, the pre-trained BERT model is fine-tuned using a small number of labelled data. The experiments show that the proposed method achieves optimal performance in completing the knowledge graph of the Chinese text of cultural relics.

The contributions of this work are described as follows:

This paper proposes a framework based on Bidirectional Encoder Representations from Transformers for Knowledge Graph Completion named BERT-KGC. The method uses a small number of labelled data to fine-tune the pre-trained BERT model to capture the rich context information of the Chinese text of cultural relics and alleviate the problem of the lack of labelled data in the field of cultural relics. To the best of our knowledge, this is the first work using a pre-trained contextual language model for the task of completing the knowledge graph in the field of Chinese cultural relics.

This paper integrates entity-type information as a part of the input of the proposed BERT-KGC model to achieve more effective representations of the entities in the knowledge graph completion task. The integration of entity-type information enables the model to obtain entity semantic information better and partly reduce the degree of ambiguity of entities and relations.

Preliminary experiments are executed using the CCR20 dataset with baseline models and the proposed BERT-KGC models. The experimental results show that the BERT-KGC model using a small quantity of labelled data to fine-tune the pre-trained language model can achieve more effective performance than that of the baseline models in the triple classification, link prediction and relation prediction tasks.

The remainder of the paper is organized as follows. Related work on embedding models, neural network-based models, language models, and multisource information incorporation for knowledge graph completion is reviewed in Section 2, and the detail of the BERT language model using entity-type information for knowledge graph completion is introduced in Section 3. In Section 4, the dataset, baselines, experimental settings and results are reported, and this is followed by the discussion in Section 5. The conclusions regarding the major research findings and future work are described in Section 6.

## 2. Related Work

Although knowledge graphs can provide high-quality structured data, large-scale knowledge graphs are still far from being complete. This problem motivates the knowledge graph completion task. Triple-based embedding models for knowledge graph embedding have been reviewed by Nguyen, D.Q. [20]. Early research focused on two approaches, which included embedding models and neural network-based models. In recent years, due to the excellent performance of language models in natural language processing tasks, especially the BERT, many studies have utilized the BERT language model to perform the knowledge graph completion task.

### 2.1. Embedding Models for Knowledge Graph Completion

Embedding models use distributed vectors to represent entities and their relations and to measure the relations between entities based on the similarity of the vectors. The classic algorithm in this area is TransE [21], which was proposed by Bordes in 2013, based on knowledge representation. TransE assumes that there is an implicit vector space, and the entities and relations in the knowledge graph are expressed in low-dimensional space to obtain the continuous vector. Then, an abstract relation between two entities is mapped to a transformation relation between two vectors. The TransE algorithm performs well in reasoning and is simple to implement, but it has considerable shortcomings in one-to-many, many-to-one and many-to-many relations.

The subsequently proposed algorithms, such as TransH [22], TransR [23], TransD [24] and TEKE [8], have improved the defects of the TransE model and achieved good results. TransH tried to solve the problem of TransE by modelling relations as hyperplanes, thereby allowing entities to play different roles in different relations. TransR simulated the entities and relations in different semantic spaces and mapped the entities from the entity space to the relational space via embedding learning. TransD, a more fine-grained model, not only considered the diversity of relations but also entities. It had fewer parameters and no matrix-vector multiplication operations. TEKE, a text-enhanced knowledge embedding model, expanded the semantic structure of the knowledge graph and each relation to own different representations for different head and tail entities to better handle 1-to-N, N-to-1 and N-to-N relations. The above methods only used the structural information observed in triples to conduct knowledge graph completion and ignored external information such as textual descriptions. Socher, R. et al. [25] proposed the neural tensor network (NTN), which represented each entity with the average word vector of its entity name and can share the textual information in similar entity names. This model could make inferences within the knowledge base to predict the relations between entities and allowed entity vectors to interact through tensors to achieve a good link prediction effect. However, the training process for this embedding method takes a long time [26]. In systems with high speed requirements, these methods still cannot meet the requirements. Moreover, the embedding models do not make full use of the word sequence information in the text. More semantic information will be lost during training, especially for complex words in natural language [27].

### 2.2. Neural Network-Based Models for Knowledge Graph Completion

In order to better apply the semantic information in text, many studies have explored applying neural networks in knowledge graph completion. Dettmers et al. [11] proposed a multilayer convolutional network model named ConvE that utilized 2D convolutions over embeddings for link prediction. The architecture of ConvE consisted of three layers: a single convolution layer, a projection layer to the embedding dimension, and an inner product layer. This model achieved good performance on common datasets. Nguyen et al. [10] proposed an embedding model named ConvKB based on a convolutional neural network that represented the three respective elements (head entity, relation, and tail entity) of each triple as a three-column matrix. They then fed these three-column matrixes to the convolutional neural network to generate different feature maps. This model achieved good performance in the link prediction task. Nguyen et al. [12] proposed CapsE to expand ConvKB by adding a capsule network layer on top of the convolution layer. CapsE achieved better performance than previous embedding models for knowledge graph completion. However, these neural network-based models need large-scale labelled datasets to attain effective performance in knowledge graph completion. Meanwhile, acquiring high-quality labelled data is time consuming and expensive for downstream applications, especially for the text of cultural relics.

### 2.3. Language Models for Knowledge Graph Completion

In recent years, studies have pre-trained the BERT language representation model with a large number of unlabelled data and then fine-tuned the pre-trained language model with an additional output layer to build state-of-the-art models for a wide range of tasks. Davison, J. et al. [28] developed an unsupervised method based on the pre-trained BERT to generate masked sentences by transforming relational triples. This method can predict the validity of triples by estimating pointwise information mutual between two entities. However, this method did not achieve good performance on the test set. Yao, L. et al. [29] proposed a framework named KG-BERT, which used pre-trained language models for knowledge graph completion. This model treated the knowledge graph completion task as a sequence classification problem and turned entities, relations and triples into textual sequences. These sequences were used to fine-tune the BERT model to predict the plausibility of a triple or a relation by computing a scoring function. KG-BERT used six benchmark English datasets to run experiments and achieve state-of-the-art performance in link prediction, triple classification and relation prediction tasks. Because the Chinese text of cultural relics has relevant context and due to the particularity of the word formation of cultural relic entities, this paper uses the BERT language model to complete the knowledge graph of Chinese cultural relics.

### 2.4. Multisource Information Incorporation for Knowledge Graph Completion

Many studies have focused on the task of integrating multisource information for knowledge representation. Wang, Z. et al. [30] used the TransE model to learn the knowledge representation in a knowledge graph incorporating textual data, and then, they used the link information in Wikipedia to make the word representation of the entities in the text as close as possible to the entities in the knowledge graph. Xie, R. et al. [31] proposed integrating the entity description information provided in a knowledge graph. After encoding, the entity description information is spliced with the vector obtained by the Translation model to obtain the final entity embeddings. Xie, R. et al. [19] proposed a model named type-embodied knowledge representation learning (TKRL) that integrates the entity-type information to learn the representation of entities and relations in the knowledge graph. They use the hierarchical-type information for the mapping matrix and for specific relation-type constraints. Their model obtained better results for the representation of knowledge graph entities and relations than previous models. Inspired by the TKRL model, this paper integrates the entity-type information into the BERT model to obtain richer knowledge representations.

## 3. Methodology

Let a knowledge base G be a collection of valid factual triples in the form of (head entity, relation, tail entity) denoted as (h,r,t), where h,t∈E, r∈R, E is a collection of entities and R is a collection of relations. Knowledge graph completion models aim to define the score function f, giving an implausibility score for each triple (h,r,t) to determine the validity of triples, where valid triples receive higher scores than invalid triples.

### 3.1. Bidirectional Encoder Representations from Transformers (BERT)

BERT, as a language representation model, uses unlabelled text to pre-train deep bidirectional representations via a masked language model, where the cost of the corpus is greatly reduced by the pre-training using unlabelled data. Then, next-sentence prediction methods capture masked and sentence-level representations, respectively, to make the model understand the relations between the two sentences. BERT is composed of a multilayer bidirectional transformer, each layer of which uses a multi-head attention mechanism to fuse the contextual information around the word and establish the strength (weight) of the connection between words. Simple linear models can be superimposed directly on the top layer of BERT for specific tasks to complete various downstream NLP tasks (e.g., text classification) without substantial task-specific architectural modifications. BERT has a simple concept and a strong ability to express words, and thus, it performs excellently in both word-level (e.g., question-and-answer tasks) and sentence-level NLP tasks (e.g., general language understanding tasks).

### 3.2. BERT-KGC Model with Entity-Type Information

In order to alleviate the scarcity of labelled data in the field of cultural relics and make full use of rich contextual information, this paper applies the BERT language model for knowledge graph completion in the field of cultural relics. Furthermore, the entity type, as a special attribute of an entity, can enrich the semantics of entities. Therefore, the entity-type information in triples is fused. When dealing with the task of completing the knowledge graph of Chinese cultural relics, the BERT-based Chinese model trained by Google is adopted since its performance in the open domain exceeds the results of the current mainstream models.

**Figure 1:**
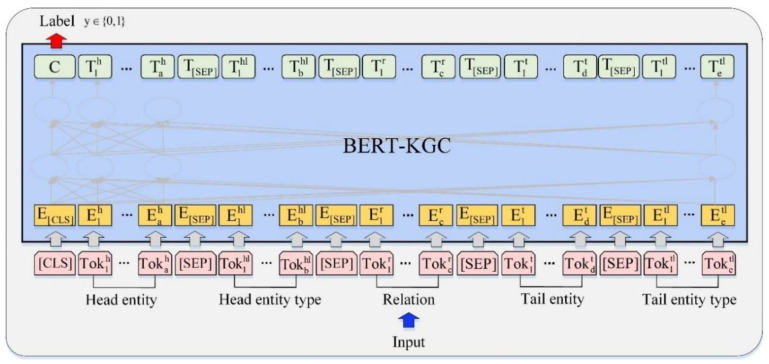
Overall fine-tuning procedures for BERT-KGC.

Inspired by the KG-BERT [29] model, which completes knowledge graphs using BERT, this paper proposes a BERT-KGC framework that is similar to KG-BERT to model the triples, as shown in Figure 1. The head entity, head entity type, relation, tail entity and tail entity type are represented as a sequence; the sentences formed by the five sequences are taken as the downstream input of BERT and the pre-trained model is fine-tuned. The input of the head and tail entities can be entity description sentences or entity names themselves.

Each input sequence always starts with a special classification label (CLS) and contains five sentences packed together into a single sequence. The first sentence represents the head entity containing tokens ${\mathrm{Tok}}_{1}^{h}$,…,${\mathrm{Tok}}_{a}^{h}$, e.g., “Afterglow-style ‘Caifeng Mingqi’ seven-stringed guqin was made in the second year of Kaiyuan of Tang Dynasty (714)” or “Afterglow-style ‘Caifeng Mingqi’ seven-stringed guqin”. The second sentence represents the type of the head entity containing tokens ${\mathrm{Tok}}_{1}^{\mathrm{hl}}$,…,${\mathrm{Tok}}_{b}^{\mathrm{hl}}$, e.g., “cultural relic”. The third sentence represents the relation containing tokens ${\mathrm{Tok}}_{1}^{r}$,…,${\mathrm{Tok}}_{c}^{r}$, e.g., “made in” or “collected in”. The fourth sentence represents the tail entities containing tokens ${\mathrm{Tok}}_{1}^{t}$,…,${\mathrm{Tok}}_{d}^{t}$, e.g., “Silver Censer with Openwork Design of Grapes and Flying birds is collected in Shaanxi History Museum” or “Shaanxi History Museum”. The fifth sentence represents the type of the tail entity containing tokens ${\mathrm{Tok}}_{1}^{\mathrm{tl}}$,…,${\mathrm{Tok}}_{e}^{\mathrm{tl}}$, e.g., “museum”. The five sentences are also separated with a special token (SEP) respectively.

**Figure 2:**
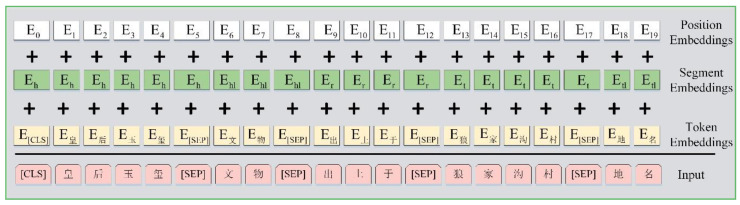
The input representation of BERT-KGC.

For a given token, its input representation is constructed by summing the corresponding token, segment and position embeddings. The input sequence representation can be visualized as in Figure 2.

Token Embeddings. For Chinese text in the field of cultural relics, Chinese words are made up of characters and beyond counting. When the words entered into the model are represented as one-hot codes, the dimensions will be very big. Meanwhile, the Chinese characters commonly used are probably more than 4000, and they are limited. Thus, this paper uses the character embeddings as token embeddings of the model. Each input character will be expressed as a vector $E_{c}$.

Segment Embeddings. The tokens in different sentences have different segment embeddings. In the head entity sentence, the tokens have the segment embedding $E_{h}$; in the relation sentence, the tokens have the segment embedding $E_{r}$; in the tail entity sentence, the tokens have the segment embedding $E_{t}$; and in the head and tail entity-type sentences, the tokens have the segment embeddings $E_{hl}$ and $E_{tl}$, respectively.

Position Embeddings. The multiple attention mechanism in Transformer cannot encode the sequential nature of the input sequence. In order to understand the sequential information of each character in the sequence, this paper sets a position embedding corresponding to each word’s position. Different tokens in the same position i∈{1,2,3,…,512} have the same position embedding. A vector is learned at each position to encode the sequence information so that the model can learn the sequential characteristics of the input.

Finally, character embedding, segment embedding and position embedding are summed up to obtain the input representation of the model.

Each input token i, denoted as $E_{i}$, is input into the BERT-KGC, which is a multilayer bidirectional transformer encoder based on the original implementation described in Vaswani et al. [32]. The final hidden vector of the special (CLS) token is denoted as $C\in R^{H}$, and the final hidden vector for the *i*_*th* input is denoted as $T_{i}\in R^{H}$, where H is the hidden state size of the pre-trained BERT. The final hidden vector $C\in R^{H}$ corresponding to the first input token (CLS) is used as the aggregate sequence representation for computing triple scores. The only new parameters introduced during triple classification fine-tuning are the classification layer weights $W\in R^{2\times H}$. The scoring function for a triple T=(h,r,t) is as follows:

(1) $$S_{T}=f\left(h,r,t\right)=\mathrm{sigmod}\left(CW^{T}\right),\;$$

where $S_{T}\in R^{2}$ is a two-dimensional real vector with $S_{T0},S_{T1}\in \left[0,1\right]$ and $S_{T0}+S_{T1}=1$.

This paper uses the cross-entropy loss function to optimize the training process for the model to obtain a model with more accurate results:

(2) $$ℒ=-\underset{T\in \left\{G\cup^{\;}G^{\prime }\right\}}{\sum }(y_{T}\log\left(S_{T0}\right)+\left(1-y_{T}\right)\log\left(S_{T1}\right)),\;$$

where $y_{T}\in \left[0,1\right]$ is the label (valid or invalid) of that triple, and T and T′ are valid and invalid triple sets, respectively. The invalid triple set T′ is the negative sample set in which the head entity or tail entity is randomly replaced by another entity; i.e., the invalid triple set T’ is simply generated by replacing the head entity h or tail entity t in a valid triple (h,r,t) ∈T with a random entity h′ or t′. T′ is structured as follows:

(3) $$\begin{array}{c} T^{\prime }=\left\{\left(h\prime ,r,t\right)|h\prime \in E\wedge h\prime \neq h\wedge \left(h\prime ,r,t\right)\notin T\right\}\\ \cup^{\;}\left\{\left(h,r,t^{\prime }\right)|t\prime \in E\wedge t\prime \neq t\wedge \left(h,r,t^{\prime }\right)\notin T\right\}\\ \cup^{\;}\left\{\left(h,r^{\prime },t\right)|r\prime \in R\wedge r^{\prime }\neq r\wedge \left(h,r^{\prime },t\right)\notin T\right\}\; \end{array}$$

where E and R are the set of entities and the set of relations. In particular, if the triples (h′,r,t),$\;\left(h,r^{\prime },t\right)$ or (h,r,t′) are already in the valid triple set T′, they will not be treated as an invalid example.

## 4. Experiments

### 4.1. Dataset and Baselines

#### 4.1.1. Dataset

To evaluate the proposed method, a knowledge graph dataset of the Chinese text of Cultural Relics called CCR20 is constructed under the guidance of cultural relic experts and museum specialists. In CCR20, entities are obtained in two ways. One way is to extract the entities from the structured data of the Shaanxi History Museum (http://www.sxhm.com/) and the List of National Cultural Relics Collection (LNCRC, http://gl.sach.gov.cn/#/public-service), including three types of cultural relics: pottery, porcelain and bronzeware. Another way is to automatically extract entities from semi-structured and unstructured data, provided by Wikipedia and online museums (http://www.chnmuseum.cn/), using the entity extraction method proposed by Zhang et al. [33]. The relation types and entity types are determined by the guidance of cultural relic experts. This paper uses the relation extraction method proposed by Zhang et al. [34] to extract the relations between two entities and construct the triplets using the entities and relations between two entities. In the experiments, invalid triples are generated by replacing an element of a valid triple with another randomly selected element. Then, the entities and triples are given to cultural relic experts and museum specialists for further supplementation and calibration. Finally, a more comprehensive and accurate dataset is obtained. Table 1 provides the detailed summary statistics of CCR20.

#### 4.1.2. Baselines

To validate and test the effectiveness of the proposed model BERT-KGC, the following baseline models will be compared with the proposed model KGC-BERT using the CCR20 dataset:

- TransE, the classical link embedding model proposed by Bordes et al. [21].
- TransH, an extension of TransE [22].
- TransR, an extension of TransE [23].
- TransD, an improvement of TransR [24].
- TEKE, a knowledge graph representation learning method taking advantage of the rich context information in a text corpus [8].
- NTN, the neural tensor network proposed by Socher et al. [25].
- TKRL, knowledge graph embeddings with hierarchical entity types and constraint information between entity types and relations [19].
- ConvE, based on the CNN model proposed by Dettmers et al. [11].
- ConvKB, an extension of ConvE proposed by Nguyen et al. [10].
- CapsE, which added a capsule network layer on top of the convolution layer, proposed by Nguyen et al. [12].
- KG-BERT, a knowledge graph completion framework based on pre-trained language models, which was proposed and applied to English text by Yao et al. [29].

### 4.2. Experimental Settings

This paper chooses the pre-trained BERT-Base Chinese model and uses Chinese simplified traditional language with 12 layers, 12 heads, total parameters = 110 M and H = 768 to initialize BERT-KGC. According to the preliminary experimental results, this paper sets the hyper-parameters for BERT-KGC for fine-tuning as follows: batch size: 32, activation function: GELU, learning rate (Adam): 5 × 10^−5^, and dropout rate: 0.1. Other parameter values were also tested in the model, and the results were not much different. The number of epochs is changed for different tasks: 3 for triple classification, 5 for link prediction and 20 for relation prediction. In the preliminary experiments, the relation prediction task provided better results after more epochs, whereas the other two tasks required only a few epochs to obtain good results.

### 4.3. Experimental Results

#### 4.3.1. Performance Comparison of BERT-KGC and the Baselines in Triple Classification

The triple classification task judges whether a given triple (h, r, t) is correct or not. This study conducted a series of comparative experiments to identify the effectiveness BERT-KGC in the triple classification. The triple classification results of different methods for the dataset CCR20 are presented in Table 2. In this paper, the precision (P), recall (R) and F1-score (F1) of the triple classification are used to measure the effectiveness of these models.

Table 2 shows that methods based on the translation model (e.g., TransE, TransH, TransR, TEKE and TransD) have relatively low results for precision, recall and F1-score that are more than 10% lower than those for the BERT-KGC model. Perhaps they do not make better use of the hierarchical information between relations and entities. NTN and deep models (e.g., ConvE, ConvKB and CapsE) achieve relatively better results than the translation models, but the results are still lower than for BERT-KGC. Perhaps they effectively leverage the deeper relations of the text, but they can only detect a very limited amount of content and have to overlay many layers to capture long-term information. TKRL with entity hierarchical types achieved relatively high results; thus, perhaps entity hierarchical types play a role in enriching entity semantics. KG-BERT(a) obtained the second-best precision, F1-score and recall, mainly because it used the BERT language model with the multi-head attention mechanism that can extract multiple semantics. The results of the proposed model BERT-KGC are considerably higher than the results of all the baseline models, which shows that using the entity, entity types and relations as the input sequence of the model allows BERT-KGC to better capture the word-forming features of cultural relic entities.

#### 4.3.2. Performance Comparison of BERT-KGC and the Baselines for Link Prediction

Link prediction replaces the head entity h given (?, r, t) or the tail entity t given (h, r, ?) with the entities in the entity set, calculates the scores for all triples, and then obtains the rank of the original triplet among all the triples. The link prediction results are evaluated using the scoring function to rank the results for test triples. This study conducted some comparative experiments to verify the effectiveness of link prediction. Some classical baseline models were tested with the OpenKE toolkit (Han et al. 2018) (https://github.com/thunlp/OpenKE), and the other baseline comparison results were obtained from the original papers. The performance comparison of the different methods for the CCR20 dataset is presented in Table 3.

This paper reports two common metrics, the mean rank (MR) and Hits@10. A lower MR is better, while a higher Hits@10 is better. The MR of BERT-KGC is 897, which is the lowest MR among the various models. The Hits@10 of BERT-KGC is 52.5%, which is slightly better than the results for baseline models such as the translation models and deep models. BERT-KGC performs better than the closely related model KG-BERT in MR and HITS@10. BERT-KGC gains significant improvements of 1136 − 897 = 239 in MR (about a 21% relative improvement) and also obtains a 53.5 − 52.3 = 1.2 relative improvement in Hits@10. The BERT-KGC model has better MR and Hits@10 performance on the CCR20 cultural relic dataset. Perhaps, the method benefits from the different attention of the BERT-KGC that focuses on different semantics to better discover more important information between entities and relations. Moreover, BERT-KGC integrating the entity-type information can enrich the semantics of entities and reduce the degree of ambiguity of entities and relations to some degree.

#### 4.3.3. Performance Comparison of BERT-KGC and the Baselines for Relation Prediction

The relation prediction task is predicting the missing relations r given (h, ?, t) in triples. This paper still reports the scoring function, similar to link prediction, to evaluate the results of relation prediction. The mean rank (MR) and Hits@10 are also used to evaluate the relation ranking. The performance comparison of the different methods for the CCR20 dataset is presented in Table 4.

Table 4 shows that BERT-KGC obtains significant improvements of 387 − 98 = 289 in the MR. In particular, BERT-KGC has an MR that is 132 − 98 = 34 higher than that of KG-BERT(b), which is about a 26% relative improvement. Specifically, the BERT-KG model achieves the highest performance in Hits@10, with relative improvements of 2.7–6.6% over the baselines. Perhaps, this is because BERT-KGC pays attention to different semantics in order to better find more important information from entities and relations. In addition, BERT-KGC integrates entity-type information, which can enrich the semantics information of entities and reduce the ambiguity of entities and relations to a certain extent, so it is slightly better than the baseline models.

#### 4.3.4. The Influence of Training Data Proportions on Triple Classification

The BERT language model captures the deep language information of the text in the unsupervised pre-training stage. In the downstream tasks, only a small number of labelled data are needed to fine-tune the pre-trained model to achieve good performance.

**Figure 3:**
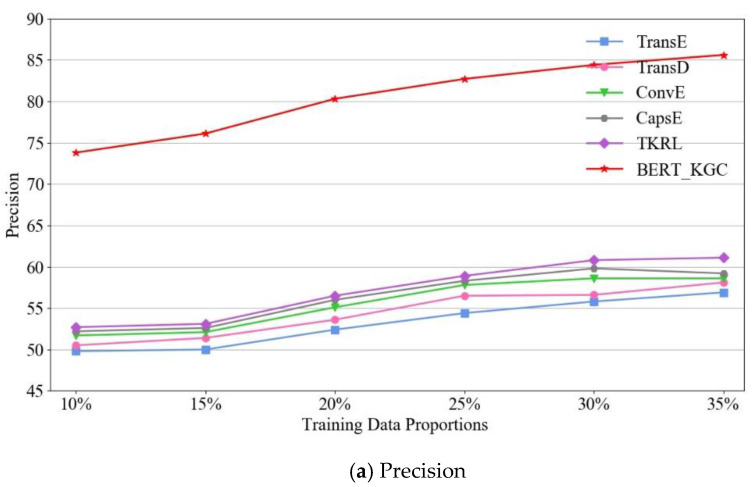

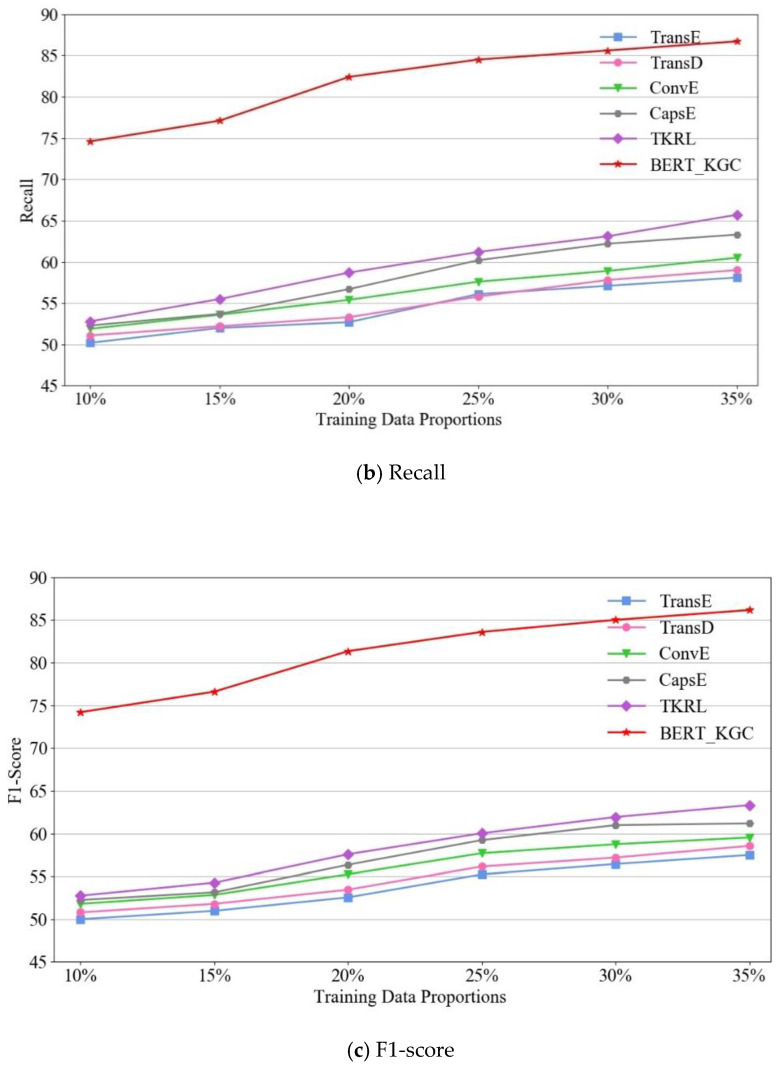
The influence of the training data proportions on the triple classification (%). Figure (**a**–**c**) is the precision, Recall and F1-score of the triple classification (%) respectively.

To verify the influence of the training data proportions on the triple classification, this paper, respectively, uses 10%, 15%, 20%, 25%, 30% and 35% of the triples of the CCR20 dataset as the initial training dataset to train the multiple baseline models and the BERT-KGC model. Figure 3 shows the triplet classification results.

The results in Figure 3 show that the results for the baseline models are relatively low when fewer training data are used. Meanwhile, the precision, recall and F1-score of the BERT-KGC model are 80.3, 82.4 and 81.34, respectively, when using 20% of the data as training data to fine-tune the model, and the precision, recall and F1-score are 85.6, 86.7 and 86.2, respectively, when using 35% of the data as training data, which is pretty close to the best result of BERT-KGC. These encouraging results suggest that BERT-KGC learned better textual features through a deep model, made full use of the rich language patterns in a large number of external text data to overcome the sparsity of the knowledge graph and enhanced the generalization ability of the model.

## 5. Discussion

Due to the lack of labelled data for the Chinese cultural relic knowledge graph and the particularity of the word formation of Chinese cultural relic entities, the existing knowledge graph completion methods are ineffective. This paper proposes a model named BERT-KGC based on the BERT language representation model with entity-type information for the knowledge graph completion of the Chinese texts of cultural relics. In the triple classification, link prediction and relation prediction tasks, the proposed BERT-KGC model achieves better performance than multiple baselines, which suggests that the BERT-KGC model based on the BERT language representation model integrating the entity-type information can extract multiple semantics using the multi-head attention mechanism and can enrich the semantics of entities using entity-type information. The main hypothesis for the good performance of the model BERT-KGC on dataset CCR20 is that the dataset contains entities with very high type-distinguishing indegrees. Examples include, the entities “Tiger-shaped Tally” with type “Bronze” and “Silver Censer with Openwork Design of Grapes and Flying birds” with type “Silver”, whose different types of information have high type-distinguishing indegrees. The successful modelling of such high indegree entities requires capturing this information. The model BERT-KGC takes the type as part of the input and better captures the type information. Meanwhile, this paper uses different summations of token, segment and position embeddings to represent the head entity, head entity type, relation, tail entity and tail entity type, respectively, in a triple as input tokens for the model. This allows the model to make a good distinction between the semantic information of different entities and the relations between them, which suggests the usefulness of considering different segment embeddings of the head entity and tail entity. In addition, BERT-KGC only uses a small number of labelled data to fine-tune the pre-trained model, achieve good performance and alleviate the problem of scarce high-quality labelled data in the field of cultural relics.

## 6. Conclusions and Future Work

This paper proposes a knowledge graph completion framework termed BERT-KGC for the Chinese text of cultural relics. BERT-KGC uses the BERT pre-trained language model with entity-type information. This paper treats entities, entity types and relations as textual sequences, while considering the knowledge graph completion task as a classification task. To evaluate our proposed method, we construct a knowledge graph dataset of the Chinese text of cultural relics called CCR20 using the guidance of cultural relic experts and museum specialists. The results of the preliminary experiment using the baseline models and the BERT-KGC model on the CCR20 dataset demonstrate that the proposed BERT-KGC model integrating the entity-type information can reduce the degree of ambiguity of entities and relations to some degree, make more effective use of the rich contextual language information of the Chinese text of cultural relics and obtain higher prediction results than multiple baselines. Moreover, our method uses a small number of labelled data to fine-tune the pre-trained BERT model to alleviate the problem of the lack of labelled data in the field of cultural relics. To our knowledge, this is the first work applying the BERT language model to the task of completing the knowledge graph of cultural relics, particularly in the field of Chinese cultural relics. This indicates a prospective strategy of expanding the triple embedding models to improve the ranking quality of knowledge graph completion and provide a technical basis for the sharing of information resources on cultural relics and their protection.

The limitation of this paper is that the model was not extended to a deeper level of application. In subsequent studies, the model level will be extended to groups to be applied to the task of knowledge graph completion. In addition, this paper adopted the static knowledge graph completion method to complete the knowledge graph of cultural relics and achieve effective performance. In future work, the language model will be utilized to further research dynamic knowledge graph completion. This is a more meaningful and interesting future direction to explore.

## Figures and Tables

**Table 1:** Summary statistics of CCR20.

Dataset	# Rel	# Ent	# Train	# Dev	# Test
CCR20	16	34,877	69,642	2908	3069

**Table 2:** Performance comparison of BERT-KGC and the baselines for triple classification.

Method	P	R	F1
TransE (Bordes et al. 2013) [21]	70.3	72.2	71.2
TransH (Wang et al. 2014) [22]	72.3	70.4	71.3
TransR (Lin et al. 2015b) [23]	75.5	77.6	76.5
TEKE (Wang and Li 2016) [8]	75.8	78.3	77.1
NTN (Socher et al. 2013) [25]	76.0	79.4	77.7
TransD (Ji et al. 2015) [24]	76.7	80.9	78.7
ConvE (Dettmers et al. 2018) [11]	77.3	81.6	79.4
ConvKB (Nguyen et al. 2017) [10]	77.9	84.3	81.1
CapsE (Nguyen et al. 2019) [12]	80.4	84.4	82.3
TKRL (Xie et al. 2016) [19]	81.1	85.0	83.3
KG-BERT(a) (Yao et al. 2019) [29]	83.8	86.6	85.2
BERT-KGC	**86.3**	**88.7**	**87.5**

Note: The experimental results are for BERT-KGC and baseline methods in triple classification. The results are in percentages. The baseline comparison results were obtained from the original papers. Bold denotes the best result, while the second-best score is underlined.

**Table 3:** Performance comparison of BERT-KGC and baselines for link prediction.

Method	MR	Hits@10
TransE (Bordes et al. 2013) [21]	2594	47.3
TransH (Wang et al. 2014) [22]	2965	46.2
TransR (Lin et al. 2015b) [23]	3272	48.4
NTN (Socher et al. 2013) [25]	3315	47.2
TransD (Ji et al. 2015) [24]	3303	47.3
ConvE (Dettmers et al. 2018) [11]	3298	49.3
ConvKB (Nguyen et al. 2017) [10]	2592	50.3
CapsE (Nguyen et al. 2019) [12]	2945	51.3
TKRL (Xie et al. 2016) [19]	2108	51.9
KG-BERT(a) (Yao et al. 2019) [29]	1136	52.2
BERT-KGC	**897**	**53.5**

Note: MR denotes the mean rank of the correct entities. Hits@10 is the proportion of correct entities in the top 10 reported in %. Bold denotes the best result, while the second-best score is underlined.

**Table 4:** Performance comparison of BERT-KGC and the baselines for relation prediction.

Method	MR	Hits@10
TransE (Bordes et al. 2013) [21]	379	78.2
TransH (Wang et al. 2014) [22]	370	79.4
TransR (Lin et al. 2015b) [23]	369	78.9
NTN (Socher et al. 2013) [25]	387	77.6
TransD (Ji et al. 2015) [24]	332	79.1
ConvE (Dettmers et al. 2018) [11]	259	79.4
ConvKB (Nguyen et al. 2017) [10]	264	80.6
CapsE (Nguyen et al. 2019) [12]	260	81.8
TKRL (Xie et al. 2016) [19]	246	80.2
KG-BERT(b) (Yao et al. 2019) [29]	132	82.1
BERT-KGC	**98**	**84.8**

Note. Bold denotes the best result, while the second-best score is underlined.
